# Association between baseline psychological attributes and mental health outcomes after soldiers returned from deployment

**DOI:** 10.1186/s40359-017-0201-4

**Published:** 2017-10-05

**Authors:** Yu-Chu Shen, Jeremy Arkes, Paul B. Lester

**Affiliations:** 1 Graduate School of Busines and Public Policy, Naval Postgraduate School, Monterey, CA 93943 USA; 2 National Bureau of Economic Research, Cambridge, MA 02138 USA; 3 Research Facilitation Laboratory, US Army, Monterey, CA 93940 USA

**Keywords:** PTSD, Depression, Psychological attributes, Resilience, military, Public safety sector

## Abstract

**Background:**

Psychological health is vital for effective employees, especially in stressful occupations like military and public safety sectors. Yet, until recently little empirical work has made the link between requisite psychological resources and important mental health outcomes across time in those sectors. In this study we explore the association between 14 baseline psychological health attributes (such as adaptability, coping ability, optimism) and mental health outcomes following exposure to combat deployment.

**Methods:**

Retrospective analysis of all U.S. Army soldiers who enlisted between 2009 and 2012 and took the Global Assessment Tools (GAT) before their first deployment (*n* = 63,186). We analyze whether a soldier screened positive for depression and posttraumatic stress disorder (PTSD) after returning from deployment using logistic regressions. Our key independent variables are 14 psychological attributes based on GAT, and we control for relevant demographic and service characteristics. In addition, we generate a composite risk score for each soldier based on the predicted probabilities from the above multivariate model using just baseline psychological attributes and demographic information.

**Results:**

Comparing those who scored in the bottom 5 percentile of each attribute to those in the top 95 percentile, the odds ratio of post-deployment depression symptoms ranges from 1.21 (95% CI 1.06, 1.40) for organizational trust to 1.73 (CI 1.52, 1.97) for baseline depression. The odds ratio of positive screening of PTSD symptoms ranges from 1.22 for family support (CI 1.08, 1.38) to 1.51 for baseline depression (CI 1.32, 1.73). The risk profile analysis shows that 31% of those who screened positive for depression and 27% of those who screened positive for PTSD were concentrated among the top 5% high risk population.

**Conclusion:**

A set of validated, self-reported questions administered early in a soldier’s career can predict future mental health problems, and can be used to improve workforce fit and provide significant financial benefits to organizations that do so.

**Electronic supplementary material:**

The online version of this article (10.1186/s40359-017-0201-4) contains supplementary material, which is available to authorized users.

## Background

The U.S. Department of Defense is the largest employer in the world with over 3.2 million employees, and while its workforce is typically younger and in better physical health than most, it is hardly immune to the effects of psychological health disorders on its workforce. In fact, the DoD’s mission – to provide for the common defense of the United States – likely exacerbates the prevalence of psychological health problems because service members regularly face significant stressors such as combat trauma and extended separation from family members [1]. Moreover, most service members eventually leave the military and integrate into the civilian workforce, and those service members suffering from mental health disorders carry this burden with them into their civilian life and workplace.

Given that the U.S. military has served in combat operations in Iraq and Afghanistan for nearly 15 consecutive years, it is not at all surprising that recent research has documented an increasing trend of psychological health service needs amongst service members. For example, rates of post-traumatic stress disorder (PTSD) among those returning from service in Iraq and Afghanistan have ranged from 5 to 45%, depending on the studied population and how PTSD is measured [2, 3], while rates of depression range from 14 to 20% [4–7]. Moreover, there is substantial evidence that the rates of major mental health problems are on the rise and have been associated with mounting costs in terms of treatment and lost productivity [3, 6, 8–11]. They are triggered both by stressors associated with combat exposure as well as stressors and difficulty adopting to civilian environments after returning from deployment [2, 12, 13].

Given the severity of the consequences of these problems for both the individuals affected and for the organization, it seems prudent to ask whether or not such problems could be mitigated before they develop. One prior study has demonstrated that pre-deployment mental health screening coupled with in-theater care coordination can significantly reduce subsequent clinical encounters for psychiatric disorders [14]. One important hypothesis that has not been fully explored in the literature is that some soldiers might enter the military with poor psychological health such that they have much higher demand for mental health services when they are exposed to the stressors involved with the protracted war on terrorism or life stressors after returning from deployment, relative to others who are psychologically fit for the military life. We are only aware of one other study that explored similar hypothesis—in that study the authors showed that soldiers who scored high on measures of psychological strengths, such as hope, optimism, confidence, and resilience prior to a combat deployment were less likely to be diagnosed with mental health or substance abuse problems once they returned home [15]. Such insights can be used by the Army or other public safety organizations to develop strategies to recruit young workers who are fit, both physically and psychologically, or develop early interventions for those who might be at higher risk of developing costly mental health problems. Both approaches might reduce the prevalence of costly mental health problems over time. In this study, we explore one potential strategy to achieve this goal by taking advantage of the new data that captured individual soldier’s baseline psychological attributes as part of the recently initiated Comprehensive Soldier and Family Fitness (CSF2) program by the U.S. Army [16].

CSF2 was launched in 2009 in response to the rapid rise in psychological health problems in soldiers who repeatedly deployed to combat in Iraq and Afghanistan, and the program’s goals are to increase the resilience and psychological health of Army soldiers through training [17, 18]. A major component of the CSF2 program is the Global Assessment Tool (GAT) which is an annual resilience and psychological health assessment completed by all members of the U.S. Army and, for new recruits, the GAT is completed within a few weeks of entering military service. The GAT is a 105-question self-administered questionnaire that captures 14 attributes of psychological health and resilience that are deemed important for life in the military [19].

By combining the GAT records with other administrative data, we analyze the association between 14 baseline attributes and U.S. Army soldiers’ probability of screening positive for two costly mental health illness—depression and PTSD —following their first combat deployment. Knowing how well these psychological attributes can predict future mental health outcomes can potentially aid the DoD in identifying a workforce that is better suited for the stresses associated with its unique environment, and provide more targeted interventions to sub-populations at greater risk for developing psychological health problems. Such a strategy can also be applicable to other organizations that share similar occupational hazards and stressful environments, such as fire, police, and other public safety departments.

## Methods

### Data and study population

We used three sources of data provided by the Army: individuals’ item-level responses to soldiers’ Global Assessment Tools (GAT); the Pre- and Post-Deployment Health Assessments; and the Army’s master personnel database containing demographic and service characteristics.

The Post Deployment Health Assessment (PDHA) is used to assess the soldiers’ state of health after a deployment in support of military operations and to assist healthcare providers in identifying present and future medical care needs [10, 20]. All soldiers who deployed are required to complete the assessment, which is administered by a trained health care provider within 30 days of returning home from a combat deployment. We focused our attention on the screening questions for depression and PTSD, as well as questions measuring each soldier’s level of combat exposure (described in more detail below). For 63% of our sample, we also were able to match their PDHA records to their pre-deployment health assessment. We used the pre-deployment assessment to further control for pre-deployment psychological health status.

Our sample included all active duty Army soldiers who completed their first GAT anytime between October 2009 and March 2013 and who had a valid PDHA after their first GAT date (*n* = 223,492). In our main analysis, we further restricted the sample to those who enlisted between 2009 and 2012 and whose first deployment occurred after they took their first GAT (*n* = 63,186); this restriction ensures that the measured psychological attributes are not influenced by prior military and deployment experiences. Among this sample, the median number of days between GAT assessment date and arrival date at the combat theater is 290 days (recruits typically spend 9 months in basic and advanced trainings).

In an alternate analysis exercise, we include all soldiers. Comparing results from both the restricted sample and the whole sample allow us to investigate whether the relationship between these baseline psychological attributes and post-deployment health conditions differ whether or not a person experienced military life before taking the GAT.

### Outcome measures

We examined two mental health outcomes. First, we defined an indicator for positive screening of depression symptoms using responses to two questions: (1) Over the past month, [how much have you] had little interest or pleasure in doing things? and (2) Over the past month, [how much have you been] feeling down, depressed, or hopeless? This 2-item Patient Health Questionnaire (PHQ-2) was modified from a validated instrument widely used in primary care settings [9, 21–23]. Consistent with Army’s mental health referral guideline, a soldier is at risk of clinical depression if he answered “half the days” or “nearly every day” on either question [20, 21].

Second, we defined an indicator for positive screening of PTSD symptoms using responses to the Primary Care PTSD screen (PC-PTSD) [24] within the PDHA. The PC-PTSD, based on DSM-IV version of PTSD, consists of four screening questions identifying whether the soldier experiences the following conditions: feeling constantly on guard, avoiding situations that remind him or her of the traumatic event, having nightmares as if reliving the traumatic event, and feeling detached. These questions correspond to the three symptom clusters of PTSD and have good diagnostic efficiency [24, 25]. Consistent with prior literature and Army’s health referral guideline, a soldier screens positive for PTSD symptoms if he/she responds positively to at least two of the four screening questions [9, 10, 26–30].

### Measures of psychological attributes

We focus our discussion below on the relationship between individual GAT responses and the aggregated psychological attributes, and refer readers to other reports for complete GAT details [31–33]. Responses to the 105 GAT questions are collected as either binary responses or on a five- or 10-point Likert scale. We first standardized individual questions to be within a scale of one to five. For binary responses, we converted the no and yes responses to 1 and 5 point, respectively. We then aggregated responses to these individual questions into 14 psychological attributes, and define the GAT score for each attribute as the average of the individual item responses. Each attribute is based on previously validated instruments: depression [34]; catastrophizing [35]; positive affect [36]; adaptability [37]; coping ability [38]; optimism [39]; character [40]; family satisfaction and family support [36]; engagement in the workplace [40, 41]; friendship [36]; inclusion [42]; organizational trust [43–45]; and spiritual fitness [46]. We provide the actual questions for each attribute in Additional file 1: Table S1.

Each of these attributes were designed to be predictive of mental health outcomes within the context of military settings. For example, attributes such as optimism and catastrophizing reflect how a person might respond to combat stressors. Positive affect and organizational trust capture how soldiers respond to trust in leadership, and violations of trust can precipitate psychological health problems. Lastly, attributes that capture resilience (such as spirituality, coping ability) and external support (such as family support, friendship, inclusion) could reflect external psychological resources that are available to the soldier. Consequently, we anticipate that each of predictors will show some relationship with mental health outcomes.

For all attributes except two, a higher scale reflects more positive psychological attributes; the two other attributes—depression and catastrophizing— are reverse-coded for consistency.

Following prior work [47], our key independent variables were the 14 binary indicators of whether a soldier scored in the bottom 5%iles for each of the 14 GAT attributes. Specifically, we created a binary indicator for each attribute that takes on the value one if a soldier’s score for that attribute is in the bottom 5 percentiles of the whole sample. We chose the 5 percentile cutoff because past research suggests that high risk people tend to concentrate in the top or bottom 5 percentile (depending on the nature of the risk factor) [47, 48]. In our sensitivity analysis, we also estimated our models using bottom 10 percentile (results available upon request), and reached similar conclusions.

### Statistical methods

We estimated logistic regression models for each psychological health outcome. In all models, we controlled for demographic and service characteristics, including gender, age, race/ethnicity, marital status, education, Armed Forces Qualification Test score, broad military occupation group (combat arm, combat service, service support, aviation, other), and indicators of a soldier’s rank. All time-varying variables (such as age, rank) are based on their value at the time of the post-deployment assessment. Additionally, we included three variables from the PDHA on self-reports of combat experiences during deployment: (1) whether the soldier witnessed deaths or dead bodies; (2) whether the soldier discharged his or her weapon; and (3) whether the soldier was wounded or in perceived danger. Lastly, as a control for baseline psychological health status, we included indicators for whether the soldier had a matching pre-deployment health assessment and whether the soldier needed psychological health counseling prior to deployment. All models were estimated using STATA version 13 [49].

Besides the main models described above, we also conducted exploratory analysis by incorporating responses from post deployment health reassessment (PDHRA) where soldiers were reassessed on the same set of psychological health outcomes 90–180 days after deployment. This exploratory analysis allows us to investigate whether our estimated odds ratios from the main model is biased due to possible delays in the onset of depression and PTSD symptoms.

## Results

Table 1 provides the descriptive statistics of the sample. The first column shows that among the 63,186 soldiers included in the main analysis, 7% screened positive for depression symptoms and 11% screened positive for PTSD. The sample is young (average age is 21.66), and mostly single (only 20% are married), reflecting the fact that our main analysis focuses on soldiers who enlisted on or after 2009 and who took GAT before their first deployment.

**Table 1:** Descriptive statistics of the restricted sample by their post-deployment mental health status

	Whole sample		None		Depression^a^		PTSD^a^	
Psychological health outcomes in PDHA								
Screen positive for depression^a^	4540	7%						
Screen positive for PTSD^a^	7012	11%						
Baseline psychological attributes (in bottom 5 percentile)								
Depression (rev. coding)	1644	3%	980	2%	563	5%	331	5%
Catastrophizing (rev. coding)	3102	5%	2144	4%	795	7%	434	6%
Positive Affect	2085	3%	1214	2%	758	7%	397	6%
Adaptability	1620	3%	1101	2%	452	4%	201	3%
Coping Ability	2564	4%	1772	4%	671	6%	333	5%
Optimism	2945	5%	1950	4%	868	8%	415	6%
Positive Character Actions	1479	2%	1004	2%	392	4%	214	3%
Engagement with Job	2256	4%	1607	3%	548	5%	283	4%
Inclusion	2153	3%	1318	3%	722	7%	379	5%
Organizational Trust	1407	2%	924	2%	390	4%	249	4%
Friendship	2330	4%	1515	3%	700	6%	373	5%
Family Satisfaction	2301	4%	1508	3%	655	6%	387	6%
Family Support	2002	3%	1354	3%	528	5%	348	5%
Spirituality	2107	3%	1447	3%	574	5%	280	4%
Combat exposure								
witnessed deaths during deployment	19,421	31%	12,824	26%	4418	41%	4535	65%
discharged weapon during deployment	11,419	18%	7656	16%	2344	22%	2802	40%
wounded or in danger during deployment	15,705	25%	9772	20%	4104	38%	4008	57%
Demographic and service characteristics								
Mean age (SD)	21.66	4.33	21.66	4.34	21.56	4.26	21.73	4.31
Male	56,597	90%	44,519	91%	9224	85%	6089	87%
Female	6589	10%	4646	9%	1611	15%	904	13%
White	40,696	64%	31,842	65%	6772	62%	4445	64%
Black	10,937	17%	8355	17%	2063	19%	1265	18%
Hispanic	8146	13%	6414	13%	1284	12%	920	13%
Asian	2910	5%	2159	4%	640	6%	310	4%
Other minority	497	1%	395	1%	76	1%	53	1%
at least college degree	6316	10%	4969	10%	1086	10%	613	9%
Single	50,640	80%	39,541	80%	8536	79%	5520	79%
Married	12,546	20%	9624	20%	2299	21%	1473	21%
Divorced	866	1%	649	1%	178	2%	114	2%
Have children	9098	14%	6928	14%	1687	16%	1131	16%
AFQT scores 31–49	21,033	33%	16,337	33%	3571	33%	2472	35%
AFQT scores 50–64	15,396	24%	11,920	24%	2660	25%	1778	25%
AFQT scores 65–92	22,062	35%	17,232	35%	3776	35%	2323	33%
Aviation	4187	7%	3292	7%	729	7%	357	5%
Combat service	3360	5%	2686	5%	570	5%	248	4%
Service support	15,332	24%	11,760	24%	2914	27%	1635	23%
Other occupation	10,630	17%	8534	17%	1706	16%	869	12%
Rank E1-E2	1857	3%	1303	3%	455	4%	284	4%
Rank E3	17,056	27%	13,207	27%	3059	28%	1880	27%
Rank E4 and above	44,273	70%	34,655	70%	7321	68%	4829	69%
pre-deployment assessment available	37,995	60%	29,031	59%	6946	64%	4483	64%
sought psychological health counseling pre-deployment	1399	2%	778	2%	521	5%	326	5%
sample	63,186		49,165		10,835		6, 993	

PTSD: screen positive for PTSD symptoms based on the Primary Care PTSD screen

^a^Depression: screened positive for depression symptoms based on 2-item Patient Health Questionnaire

The next three columns of Table 1 provide summary statistics for sub-populations of individuals with mental health outcomes: screened positive for depression, screened positive for PTSD, did not screen positive for either depression or PTSD symptoms. Those who screened positive for depression post-deployment were more likely to be in the bottom 5 percentiles of the 14 psychological attributes at the baseline compared to those who reported no psychological health symptoms. Not surprisingly, those who experienced more intense combat exposure were likely to develop depression and PTSD [50]. For example, among those without psychological health symptoms, 26% witnessed a death and 20% were wounded or in danger during their first deployment. For those who screened positive for depression, the corresponding rates were 41 and 38%, respectively; and for those who screened positive for PTSD, the rates were 65 and 57%, respectively. The remainder of the demographic and service characteristics were similar between those who did not report symptoms of mental health problems and those who did.

**Fig. 1:**
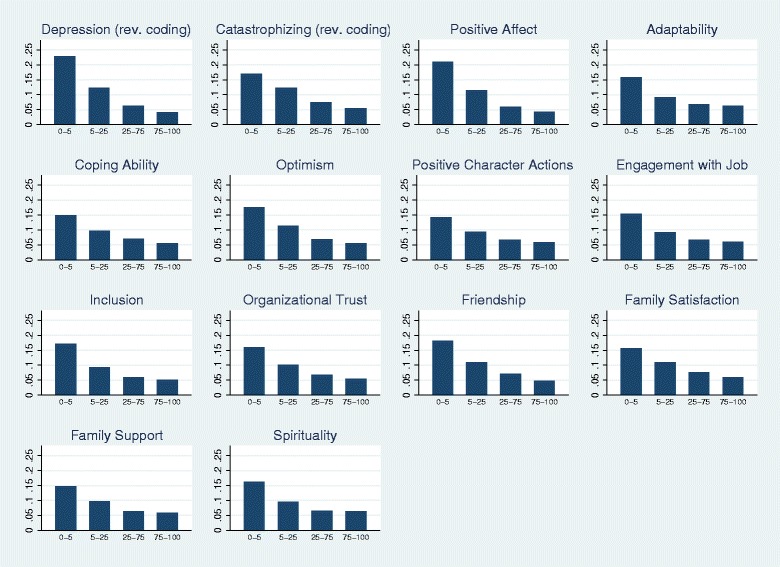
Share of soldiers in the restricted sample screened positive for depression symptology post deployment in various percentile ranges of GAT scores by attributes

To give a better sense of the relationship between the baseline attributes and the mental health outcomes, Fig. 1 shows the percentages of soldiers who screened positive for depression post-deployment within various percentile groups for the 14 baseline psychological attributes (≤ 5th percentile, 5th–25th percentile, 25th–75th, and top quartile). Using Positive Affect as an example, 20% of solders in the bottom 5 percentile of this attribute screened positive for depression after deployment, compared to less than 5 percent in the top quartile. We observed the same pattern across all 14 attributes: the post deployment depression rate was substantially higher in the lowest 5 percentiles compared to the other three percentile categories.

The first column of Table 2 shows the results of the complete logistic regressions for the depression outcome. For clarity, we only show the regression-adjusted odds ratios for the 14 psychological attributes; the complete regression results are included as Additional file 2: Table S2. Controlling for relevant demographic and service characteristics, 10 out of 14 baseline psychological attributes were significantly predictive of post-deployment depression symptoms (all at the 1% significance level, except for organizational trust, which is significant at the 5% level). For example, the odds of a soldier screening positive for depression was 1.47 (95% CI 1.27, 1.71) higher for those who scored at the bottom 5 percentile in positive affect attribute compare to those in the top 95 percentile. Among the remaining attributes that have statistically significant estimates, the odds ratio ranged from 1.19 (CI 1.00, 1.42) for organizational trust to 1.51 (CI 1.31, 1.74) for inclusion.

**Table 2:** Regression-adjusted odds ratio of post-deployment depression and PTSD on soldiers in the restricted sample

Outcome = Odds Ratio (95% CI)	Depression^*^	PTSD^*^
Baseline psychological attributes (in bottom 5 percentile)		
Depression (rev. coding)	1.47***	1.48***
	[1.25–1.72]	[1.27–1.74]
Catastrophizing (rev. coding)	1.42***	1.08
	[1.26–1.60]	[0.96–1.23]
Positive Affect	1.47***	1.27***
	[1.27–1.71]	[1.09–1.48]
Adaptability	0.96	0.93
	[0.80–1.15]	[0.77–1.11]
Coping Ability	1.00	0.94
	[0.87–1.16]	[0.82–1.08]
Optimism	1.41***	1.01
	[1.24–1.61]	[0.88–1.15]
Positive Character Actions	0.90	1.07
	[0.75–1.09]	[0.89–1.28]
Engagement with Job	1.12	0.90
	[0.96–1.30]	[0.77–1.04]
Inclusion	1.51***	1.42***
	[1.31–1.74]	[1.23–1.64]
Organizational Trust	1.19**	1.39***
	[1.00–1.42]	[1.18–1.64]
Friendship	1.47***	1.13*
	[1.28–1.68]	[0.98–1.30]
Family Satisfaction	1.38***	1.35***
	[1.21–1.58]	[1.19–1.54]
Family Support	1.37***	1.31***
	[1.19–1.59]	[1.14–1.50]
Spirituality	1.23***	1.05
	[1.06–1.44]	[0.90–1.22]
witnessed deaths during deployment	1.63***	3.18***
	[1.51–1.76]	[2.98–3.39]
discharged weapon during deployment	0.91**	1.57***
	[0.83–1.00]	[1.46–1.68]
wounded or in danger during deployment	2.22***	8.16***
	[2.04–2.42]	[7.41–8.97]
Sample size	62,913	62,754

*** *p* < 0.01, ** *p* < 0.05, * *p* < 0.1

Note:

Depression: screened positive for depression symptoms based on 2-item Patient Health Questionnaire

PTSD: screen positive for PTSD symptoms based on the Primary Care PTSD screen

Additional variables in the regression include gender, race, age, marital status, dependent quantity, rank, AFQT percentile, military occupational specialty, pre-deployment psychological health counseling need

The second column of Table 2 reports the results for PTSD. The multivariate results show that six psychological attributes were significantly predictive at the 1% significance level of higher odds of screening positive with PTSD symptoms post deployment when comparing people with similar demographic and service background: the odds ratios ranged from 1.27 for positive affect (CI 1.09, 1.48) to 1.48 for depression (CI 1. 27, 1.74).

Holding soldier’s baseline psychological attributes and other demographic and service variables constant, the odds of developing depression and PTSD was 2.22 (CI 2.04, 2.42) and 8.16 (CI 7.41, 8.97) times higher, respectively, for soldiers who were wounded or perceived grave danger during deployment compared to those who did not have this experience. The odds of depression and PTSD was 1.63 (CI 1.51, 1.76) and 3.18 (CI 2.98, 3.39) times higher, respectively, among those who witnessed death compared to those without this experience.

When we incorporated responses from PDHRA in the analysis (the re-assessment that were done 90–180 days post deployment), we captured an additional 4% of soldiers screened positive for depression and PTSD from this second assessment. Our conclusions did not change when we incorporated these additional responses into our analyses (results available upon request).

**Fig. 2:**
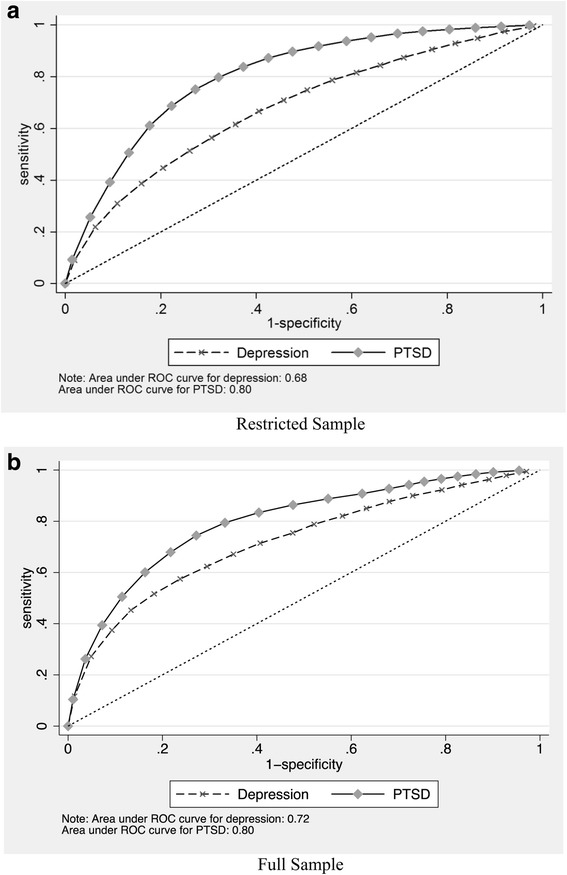
Receiver Operating Characteristic (ROC) curves

In an alternative analysis, in which we relaxed the sample restriction and include all soldiers (results included in Additional file 1: Table S1), we observed similar patterns, suggesting that the relationship between these baseline psychological attributes and post-deployment health conditions were fairly stable, and do not appear to be modified by whether a person experienced military life before taking the GAT. In the whole sample, the odds ratios were statistically significant for all 14 psychological attributes in the case of the depression outcome (OR ranges from 1.15 for family support to 2.03 for baseline depression); and 10 psychological attributes for the PTSD outcome (OR ranges from 1.12 for coping ability to 1.62 for depression). Figure 2 presents a Receiver Operating Characteristic (ROC) curve based on our model for both outcomes, and shows that the model does reasonably well in classifying soldiers into the correct outcome category (the area under the ROC curve for depression and PTSD is 0.72 and 0.80, respectively).

**Fig. 3:**
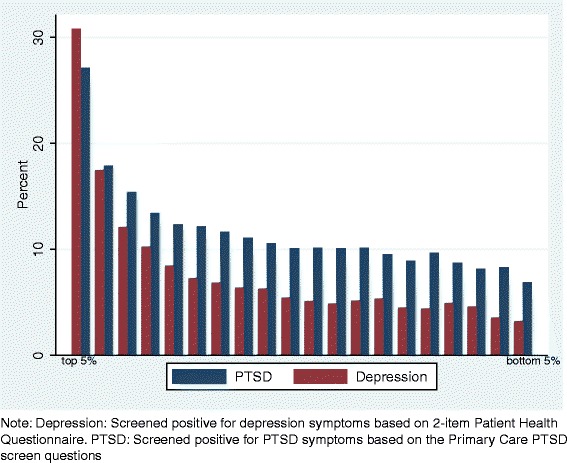
Distribution of depression and PTSD by ventiles of the predicted probabilities, based on all soldiers

Finally, we considered the possibility of using the multivariate model described above to generate a composite risk score for each soldier. Such a composite risk profile can potentially be useful in screening recruits and/or identify high risk groups for targeted intervention. In generating the risk score, we only included the baseline psychological attributes and the observable demographic information (i.e., we exclude the combat exposure variables and service characteristics as those are not observed during the recruiting stage). Based on this model, we generated the predicted probability of each outcome and then rank-ordered soldiers into 20 groups (ventiles). We plotted the fraction of each outcome across the ventiles. Given the similarities of the odds-ratio estimates between our restricted and full sample, we use the full sample for this exercise so we have sufficient number of soldiers that screened positive for each outcome to generate more stable numbers for each ventile. Figure 3 shows that 31% of those who screened positive for depression and 27% of those who screened positive for PTSD were concentrated among the top 5 percent high risk population as predicted by the model (if we were to incorporate the remaining factors from our main model in constructing the predicted probability, the concentration of risk increased to 33% for depression and 45% for PTSD among the top 5 percent high risk group).

## Discussion

There is little question that a workforce consisting of psychologically unhealthy individuals can be costly in certain high-stress or high-physical-risk occupations. In this study, we explored the longitudinal association between a number of psychological and social attributes measured upon entry into the military and mental health outcomes following return from a combat deployment. Not surprisingly, we found that soldiers who experienced significant combat exposure (especially if they were wounded or perceived to have been in grave danger) were substantially more likely to screen positive for depression and PTSD once they returned home. Yet, perhaps most germane to the goals of our study, we found that those soldiers with the worst pre-military psychological health attribute scores – those in the bottom 5% of scores – carried much higher odds of screening positive for depression and PTSD after returning home than did the top 95%. Those soldiers who scored worst might be more susceptible to developing debilitating mental health disorders when they are later exposed to combat environments.

Our results are consistent with a recent study that showed that soldiers who scored high on psychological strength measures prior to being deployed are less likely to develop mental health problems post deployment [15]. Our findings suggest that ex-ante psychological screening, in combination with other personnel information, can provide a meaningful way to either select a workforce that is more suited for stressful environments and/or to identify individuals who carry significant risk for developing these psychological health disorders and design tailored training interventions to increase their psychological health states prior to exposing them to combat.

It would appear that the financial savings for effectively enacting either strategy is non-trivial given the substantial costs of treatment and lost productivity. For context, one study estimated the total economic burden of depression in the U.S. to be $83.1 billion, where 31% were direct medical costs, 62% were workplace costs, and the remaining 7% due to mortality costs [51]. Within the military, another study estimated the 2-year costs related to PTSD and depression among those deployed range from $4–6.2 billion (average cost per case ranged from $10,298 to $25,757), where 3% were due to medical cost, 55% were due to lost productivity, and the remaining 42% due to mortality [2]. These estimates underscore the importance of including psychological health as an index of suitability to serve and point to the fact that the majority of the cost borne by the military comes from lost productivity at work, and we would expect similar results if these figures were extended to the public safety occupations.

How could organizational leadership apply what we describe here to improve overall resilience and lower costs to their organization? Carrying the military example forward, suppose the U.S. Army recruits 70,000 soldiers annually (fairly typical) and assume half of those recruits will be deployed to combat at some point during their tenure. If the Army leadership were to enact a psychological health screening tool that was resistant to strategic responses and set the exclusion criteria to the worst 5% of scores per the profile we presented in Fig. 3, then the 2-year forecasted saving based on the average per case cost described previously would range from $122.5 million to $306.5 million for one cohort alone. Admittedly this strategy would entail additional cost, such as costs to increase the recruiting pool [47] and to develop effective psychological health development programs for those already employed by the organization. However, those costs likely pale in comparison to long term psychological health treatment, lost productivity at work, and other factors described in this study. Further, there are other potential savings from such screening policy not explored here when we take into account other personnel outcomes, such as: organizational attrition [47]; an increase in organizational readiness; and a decreased strain on organizational leaders charged with ensuring that those with psychological health problems receive necessary medical care and administrative attention.

There are a few notable limitations to our study. First, while the mental health outcome measures we used in the current study have been shown to have strong predictive power towards objective clinical diagnoses, we were unable to obtain actual clinical diagnosis data for PTSD and depression. However, research has repeatedly established that there is a general stigma associated with reporting psychological health illness in the military [10, 52, 53], and therefore we expect under-reporting of mental health problems in the PHDA.

Second, the PDHA is administered fairly soon after the tour, whereas psychological health problems do not usually manifest themselves until much later. Even though our conclusions did not change when we incorporated PDHRA into our analyses (which took place 3–6 months post deployment), the timing of PDHRA might still be too soon to fully capture mental health problems. For example, a recent study found that screening conducted 6–12 weeks after deployment did not predict mental health problems that occurred 10–24 months after deployment [54], so it would be critical for future studies to capture mental health problems from a longer follow-up period in order to validate our results.

Third, in order to ensure that our baseline measure of resilience and psychological health were not influenced by the person’s military/deployment experience, our sample was restricted to soldiers who entered military service in 2009 or later and who took the GAT before their first combat deployment. While GAT data used in the present study were captured very early in soldiers’ tenure, it is still possible that their experiences in the first few weeks of military service could impact both psychological health (as measured by the GAT) and likelihood of mental health problems that emerge later in their tenure. As these initial experiences could positively or negatively affect psychological health, the effect on our analysis is unclear.

Fourth, we limited our sample inclusion criterion to include only those who were deployed to combat, so the relationships we observed might be stronger or weaker for those who were not deployed to combat. Fifth, as with any variable measured with potential error, and the GAT certainly reflects a limited measure of soldier psychological health, it ignores other factors that likely contribute to mental health, such as genetic predisposition.

Lastly, it is important to recognize that the GAT in its current form is not designed to be used as a screening tool and to do so in high stakes settings where employment decisions are made would be a mistake. Rather, we use GAT data in the current study to illustrate the *potential value* for psychological health screening in public safety and national defense occupations. When taken together, the data gathered from the GAT offers us a unique opportunity to quantify the psychological health and resilience of soldiers prior to full immersion into the military and deployment to combat zones. It would be important for any future design of any screening tool to detect and minimize strategic responding, since by then the personnel know that their career progression and chance of being deployed might depend on their pre-deployment screening answers.

Ultimately, a more effective screening tool for recruiting might involve incorporating the psychological attributes with other non-cognitive information (for example, personality factors measured by the Tailored Adaptive Personality Assessment System, TAPAS) [55, 56]. Of course, screening is only one of many strategies to reduce the financial burden associated with a workforce not well-suited to the extreme stressors common in high fidelity work environments. As suggested previously, information gained from these psychological attributes can also be used for more targeted psychological health training interventions for those who need it the most. Evaluation of these screening tools and alternative approaches can provide additional insight and identify new areas of saving for the organizations within the national defense and public safety sectors.

## Conclusion

Mental health issues among working individuals are both widespread and potentially a very serious threat to organizational functioning. The current study suggests that the set of psychological attributes examined here can serve as potentially valuable predictors of these types of issues and that organizations operating in high fidelity contexts could and should incorporate such factors into both their screening and training programs. We demonstrated the potential financial savings of screening for psychological health within the workplace, but mere financial benefit to the organization is only one reason for doing so. Perhaps more importantly, our study touches on important ethical considerations for recruiting employees with low psychological health into jobs that likely carry significant risk and possible exposure to trauma. Doing so carries a triple-threat risk – risk to the individual, organization, and society – that, if realized, cannot be undone. For such jobs, early identification of high-risk workers need to be coupled with adequate psychological and social resources to help such workers better coping skills with the stressors of their workplaces.

## Additional files

Additional file 1: Table S1. Actual GAT questions and their corresponding psychological attributes. (PDF 152 kb)

Additional file 2: Table S2. Complete regression results on restricted and whole samples. (PDF 158 kb)

## References

[1] Eaton KM, Hoge CW, Messer SC, Whitt AA, Cabrera OA, McGurk D, Cox A, Castro CA (2008). Prevalence of mental health problems, treatment need, and barriers to care among primary care-seeking spouses of military service members involved in Iraq and Afghanistan deployments. Mil Med.

[2] Tanielian T, Jaycox LH. Invisible wounds of war: psychological and cognitive injuries, their consequences, and services to assist recovery: Rand Corporation; 2008.

[3] Shen YC, Arkes J, Kwan BW, Tan LY, Williams TV (2010). Effects of Iraq/Afghanistan deployments on PTSD diagnoses for still active personnel in all four services. Mil Med.

[4] Thomas JL, Wilk JE, Riviere LA, McGurk D, Castro CA, Hoge CW (2010). Prevalence of mental health problems and functional impairment among active component and National Guard soldiers 3 and 12 months following combat in Iraq. Arch Gen Psychiatry.

[5] Milliken CS, Auchterlonie JL, Hoge CW (2007). Longitudinal assessment of mental health problems among active and reserve component soldiers returning from the Iraq war. JAMA.

[6] Shen YC, Arkes J, Williams TV (2012). Effects of Iraq/Afghanistan deployments on major depression and substance use disorder: analysis of active duty personnel in the US military. Am J Public Health.

[7] Bray R, Hourani L, Olmsted K, Witt M, Brown J, Pemberton M, Marsden M, Marriott B, Vandermaas-Peeler R, Beimar B (2008). Department of Defense survey health related behaviors among active duty personnel.

[8] Operation Iraqi Freedom (OIF) Mental Health Advisory Team (MHAT-IV) Report. http://www.combatreform.org/MHAT_IV_Report_17NOV06.pdf.

[9] Hoge CW, Auchterlonie JL, Milliken CS (2006). Mental health problems, use of mental health services, and attrition from military service after returning from deployment to Iraq or Afghanistan. JAMA.

[10] Hoge CW, Castro CA, Messer SC, McGurk D, Cotting DI, Koffman RL (2004). Combat duty in Iraq and Afghanistan, mental health problems, and barriers to care. N Engl J Med.

[11] Wells TS, LeardMann CA, Fortuna SO, Smith B, Smith TC, Ryan MAK, Boyko EJ, Blazer D, Team MCS (2010). A prospective study of depression following combat deployment in support of the wars in Iraq and Afghanistan. Am J Public Health.

[12] Brewin CR, Andrews B, Valentine JD (2000). Meta-analysis of risk factors for posttraumatic stress disorder in trauma-exposed adults. J Consult Clin Psychol.

[13] Litz BT, Schlenger WE (2009). PTSD in service members and new veterans of the Iraq and Afghanistan wars: a bibliography and critique. PTSD Research Quarterly.

[14] Warner CH, Appenzeller GN, Parker JR, Warner CM, Hoge CW (2011). Effectiveness of mental health screening and coordination of in-theater care prior to deployment to Iraq: a cohort study. Am J Psychiatry.

[15] Krasikova DV, Lester PB, Harms PD (2015). Effects of psychological capital on mental health and substance abuse. Journal of Leadership & Organizational Studies.

[16] Vie LL, Scheier LM, Lester PB, Ho TE, Labarthe DR, Seligman MEP (2015). The U.S. Army person-event data environment: a military–civilian big data Enterprise. Big Data.

[17] Casey GW (2011). Comprehensive soldier fitness: a vision for psychological resilience in the U.S. Army. Am Psychol.

[18] Cornum R, Matthews MD, Seligman ME (2011). Comprehensive soldier fitness: building resilience in a challenging institutional context. Am Psychol.

[19] Peterson C, Park N, Castro CA (2011). Assessment for the U.S. Army Comprehensive soldier fitness program: the global assessment tool. Am Psychol.

[20] Enhanced post-deployment health assessment process (DD Form 2796). http://www.pdhealth.mil/treatment-guidance/deployment-health-assessments/post-deployment-health-assessment.

[21] Kroenke K, Spitzer RL, Williams JB (2003). The patient health Questionnaire-2: validity of a two-item depression screener. Med Care.

[22] Spitzer RL, Kroenke K, Williams JB (1999). Validation and utility of a self-report version of PRIME-MD: the PHQ primary care study. Primary care evaluation of mental disorders. Patient health questionnaire. JAMA.

[23] Whooley MA, Avins AL, Miranda J, Browner WS (1997). Case-finding instruments for depression. Two questions are as good as many. J Gen Intern Med.

[24] Ouimette P, Wade M, Prins A, Schohn M (2008). Identifying PTSD in primary care: comparison of the primary care-PTSD screen (PC-PTSD) and the general health Questionnaire-12 (GHQ). J Anxiety Disord.

[25] Bliese PD, Wright KM, Adler AB, Cabrera O, Castro CA, Hoge CW (2008). Validating the primary care posttraumatic stress disorder screen and the posttraumatic stress disorder checklist with soldiers returning from combat. J Consult Clin Psychol.

[26] Grieger TA, Cozza SJ, Ursano RJ, Hoge C, Martinez PE, Engel CC, Wain HJ (2006). Posttraumatic stress disorder and depression in battle-injured soldiers. Am J Psychiatry.

[27] Hoge CW, Terhakopian A, Castro CA, Messer SC, Engel CC (2007). Association of posttraumatic stress disorder with somatic symptoms, health care visits, and absenteeism among Iraq war veterans. Am J Psychiatry.

[28] Martin C (2007). Routine screening and referrals for PTSD after returning from operation Iraqi freedom in 2005. US Armed Forces Medical Surveillance Monthly Report.

[29] Vasterling JJ, Proctor SP, Amoroso P, Kane R, Heeren T, White RF (2006). Neuropsychological outcomes of army personnel following deployment to the Iraq war. JAMA.

[30] Shen YC, Arkes J, Pilgrim J (2009). The effects of deployment intensity on post-traumatic stress disorder: 2002–2006. Mil Med.

[31] Lester PB, Harms P, Herian MN, Krasikova DV, Beal SJ (2011). The comprehensive soldier fitness program evaluation. Report 3: longitudinal analysis of the impact of master resilience training on self-reported resilience and psychological health data. DTIC Document.

[32] Lester PB, McBride S, Cornum RL, Sinclair RR, Britt TW (2013). Comprehensive soldier fitness: underscoring the facts, dismanteling the fiction. Building psychological resilience in military personnel : theory and practice.

[33] Vie LL, Scheier LM, Lester PB, Seligman ME (2016). Initial validation of the US Army global assessment tool. Mil Psychol.

[34] Peterson C, Bishop M, Fletcher CW, Kaplan MR, Yesko ES, Moon CH, Smith JS, Michaels CE, Michaels AJ (2001). Explanatory style as a risk factor for traumatic mishaps. Cogn Ther Res.

[35] Watson D, Clark LA, Tellegen A (1988). Development and validation of brief measures of positive and negative affect: the PANAS scales. J Pers Soc Psychol.

[36] Lester PB, Harms P, Bulling DJ, Herian MN, Spain SM (2011). Evaluation of relationships between reported resilience and soldier outcomes. Report# 1: negative outcomes (suicide, drug use, & violent crimes). DTIC Document.

[37] Carver CS, Scheier MF, Weintraub JK (1989). Assessing coping strategies: a theoretically based approach. J Pers Soc Psychol.

[38] Scheier MF, Carver CS, Bridges MW (1994). Distinguishing optimism from neuroticism (and trait anxiety, self-mastery, and self-esteem): a reevaluation of the life orientation test. J Pers Soc Psychol.

[39] Seligman ME (2004). Character strengths and virtues: a handbook and classification.

[40] Peterson C, Park N, Seligman ME (2005). Orientations to happiness and life satisfaction: the full life versus the empty life. J Happiness Stud.

[41] Wrzesniewski A, McCauley C, Rozin P, Schwartz B (1997). Jobs, careers, and callings: People's relations to their work. J Res Pers.

[42] Russell D, Peplau LA, Ferguson ML (1978). Developing a measure of loneliness. J Pers Assess.

[43] Mayer RC, Davis JH (1999). The effect of the performance appraisal system on trust for management: a field quasi-experiment. J Appl Psychol.

[44] Mayer RC, Davis JH, Schoorman FD (1995). An integrative model of organizational trust. Acad Manag Rev.

[45] Sweeney PJ, Thompson V, Blanton H (2009). Trust and influence in combat: an interdependence model. J Appl Soc Psychol.

[46] Fetzer Institute (1999). Multidimensional measurement of religiousness/spirituality for use in health research: a report of the Fetzer Institute/National Institute on Aging working group.

[47] Cunha JM, Arkes J, Lester PB, Shen Y-C. Employee retention and psychological health: evidence from military recruits. Appl Econ Lett. 2015(ahead-of-print:1–6.

[48] Rosellini AJ, Monahan J, Street AE, Heeringa SG, Hill ED, Petukhova M, Reis BY, Sampson NA, Bliese P, Schoenbaum M, et al. Predicting non-familial major physical violent crime perpetration in the US Army from administrative data. Psychol Med. 2015:1–14. 10.1017/S0033291715001774 PMC5111361 26436603

[49] StataCorp (2013). Stata statistical software: release 13.

[50] Adler AB, Bliese PD, McGurk D, Hoge CW, Castro CA (2009). Battlemind debriefing and battlemind training as early interventions with soldiers returning from iraq: randomization by platoon. J Consult Clin Psychol.

[51] Greenberg PE, Kessler RC, Birnbaum HG, Leong SA, Lowe SW, Berglund PA, Corey-Lisle PK (2003). The economic burden of depression in the United States: how did it change between 1990 and 2000?. J Clin Psychiatry.

[52] Greene-Shortridge TM, Britt TW, Castro CA (2007). The stigma of mental health problems in the military. Mil Med.

[53] Warner CH, Appenzeller GN, Mullen K, Warner CM, Grieger T (2008). Soldier attitudes toward mental health screening and seeking care upon return from combat. Mil Med.

[54] Rona RJ, Burdett H, Khondoker M, Chesnokov M, Green K, Pernet D, Jones N, Greenberg N, Wessely S, Fear NT (2017). Post-deployment screening for mental disorders and tailored advice about help-seeking in the UK military: a cluster randomised controlled trial. Lancet.

[55] Drasgow F, Stark S, Chernyshenko OS, Nye CD, Hulin CL, White LA (2012). Development of the tailored adaptive personality assessment system (TAPAS) to support army personnel selection and classification decisions. Drasgow consulting group URBANA Il.

[56] Stark S, Chernyshenko OS, Drasgow F, Nye CD, White LA, Heffner T, Farmer WL (2014). From ABLE to TAPAS: a new generation of personality tests to support military selection and classification decisions. Mil Psychol.

