# Supplementary material for: Association between baseline psychological attributes and mental health outcomes after soldiers returned from deployment
Source: BMC Psychol. 2017 Oct 5;5:32. doi: 10.1186/s40359-017-0201-4 (PMC5628451; doi:10.1186/s40359-017-0201-4)
Supplement: Supplementary file 1 — Actual GAT questions and their corresponding psychological attributes. (PDF 152 kb) [file 40359_2017_201_MOESM1_ESM.pdf]

Appendix Table 1. Actual GAT questions and their corresponding psychological attributes

**Psychological attributes**	**Question content**	**Group Question**	**Reverse Scored**
Adaptability	I am good at changing myself to adjust to changes in my life.	How well do these statements describe you?	
	It is difficult for me to adjust to changes.		X
	I can usually fit myself into any situation.		
Catastrophizing	When bad things happen to me, I expect more bad things to happen.	Answer in terms of how you usually think.	X
	I have no control over the things that happen to me.		X
Coping	For things I cannot change, I accept them and move on.	How well do these statements describe you?	
	I control my emotions by changing how I think about things.		
	When something stresses me out, I try to avoid it or not think about it.		X
	When something stresses me out, I try to solve the problem.		
	When bad things happen, I try to see the positive sides.		
	I usually keep my emotions to myself.		X
	When something stresses me out, I have effective ways to deal with it.		
	When I am feeling upset, I keep my feelings to myself.		X
Depression	Feeling down, depressed, or hopeless	In the past four weeks, how often have you been bothered by any of the following problems?	X
	Feeling tired or having little energy		X
	Poor appetite or overeating		X
	Feeling very angry		X
	Trouble concentrating on things, such as reading the newspaper or watching television		X
Engagement	My work is one of the most important things in my life	How well do these statements describe your feelings about your job?	
	I would choose my current work again if I had the chance		
	I am committed to my job.		
	How I do in my job influences how I feel.		
Family Satisfaction	How satisfied are you with your marriage/relationship?	During the past four weeks, how have you felt about your relationship (spouse/significant other) and your family?	
	How satisfied are you with your family?		

Family Support	My family supports my decision to serve in the Army.<br>The Army meets my family’s needs.<br>The Army makes it easy for my family to do well.	Please indicate how strongly you agree or disagree with each of the following statements.	
Friendship	How many people are there who you can always count on if you have serious problems?<br>I have a best friend.<br>I am very close to my family.<br>I have someone to talk to when I feel down.<br>I have as much contact with friends and family members outside the Army as I want or need.<br>I spend time at interests or hobbies other than work.	same as above	
Inclusion	How often do you feel left out?<br>How often do you feel close to people?<br>How often do you feel part of a group?	Please be as honest as possible	X
Optimism	In uncertain times, I usually expect the best.<br>If something can go wrong for me, it will.<br>I rarely count on good things happening to me.<br>Overall, I expect more good things to happen to me than bad.	Please be as honest and accurate as you can throughout. Answer according to your own feelings, rather than how you think most people would answer.	X
Organizational Trust	I trust my fellow Soldiers in my unit to look out for my welfare and safety.<br>I think we are better trained than most other units in the company/battalion.<br>My leaders respect and value me.<br>My immediate supervisor has much knowledge about the work that needs to be done.<br>Overall, I trust my immediate supervisor.	Please indicate how strongly you agree or disagree with each of the following statements.	
Positive Affect	joyful<br>sad<br>peaceful/calm<br>hopeful<br>angry<br>guilty<br>scared/fearful<br>bored<br>love<br>proud<br>anxious/nervous	Here are a number of words that describe different feelings and emotions. How often you have felt this way during the past four weeks?	 X<br><br><br>X<br>X<br>X<br>X<br><br><br>X

Positive Character Actions	Creativity-coming up with new ideas<br>Curiosity or interest<br>Critical thinking, open-mindedness, or good judgement<br>Love of learning<br>Perspective or wisdom<br>Bravery or courage<br>Persistence<br>Honesty<br>Zest or enthusiasm<br>Love or closeness with others (friends, family members)<br>Kindness or generosity to others<br>Social skills or social awareness or street smarts<br>Teamwork<br>Fairness<br>Leadership<br>Forgiveness or mercy<br>Modesty or humility<br>Prudence or caution<br>Self-control<br>Appreciation of beauty and excellence<br>Gratitude and thankfulness<br>Hope or optimism<br>Playfulness or humor<br>Spirituality	Think about how you have acted in actual situations during the past four weeks. Please answer only in terms of what YOU actually did. Please read carefully. Select a number from 0 to 10 according to how often you showed/used the qualities listed?	
Spirituality	I am a spiritual person of dignity and worth.<br>My life has a lasting meaning.<br>I believe that in some way my life is closely connected to all humanity and all the world.<br>The job I am doing in the military has lasting enduring meaning.	Answer in terms of whether the statement describes how you actually live your life.	
