# Supplementary material for: Association between baseline psychological attributes and mental health outcomes after soldiers returned from deployment
Source: BMC Psychol. 2017 Oct 5;5:32. doi: 10.1186/s40359-017-0201-4 (PMC5628451; doi:10.1186/s40359-017-0201-4)
Supplement: Supplementary file 2 — Complete regression results on restricted and whole samples. (PDF 158 kb) [file 40359_2017_201_MOESM2_ESM.pdf]

Appendix Table 2. Complete regression results on restricted and whole samples

	Restricted Sample		Whole Sample	
Outcome=	Depression	PTSD	Depression	PTSD
Odds ratio [95% CI]				
**Baseline psychological attributes (in bottom 5 percentile)**				
Depression (rev. coding)	1.47***	1.48***	2.03***	1.62***
	[1.25 - 1.72]	[1.27 - 1.74]	[1.91 - 2.16]	[1.52 - 1.73]
Catastrophizing (rev. coding)	1.42***	1.08	1.19***	1.12***
	[1.26 - 1.60]	[0.96 - 1.23]	[1.12 - 1.26]	[1.05 - 1.18]
Positive Affect	1.47***	1.27***	1.65***	1.49***
	[1.27 - 1.71]	[1.09 - 1.48]	[1.55 - 1.76]	[1.39 - 1.59]
Adaptability	0.96	0.93	1.07*	0.94
	[0.80 - 1.15]	[0.77 - 1.11]	[0.99 - 1.15]	[0.87 - 1.02]
Coping Ability	1.00	0.94	1.17***	1.12***
	[0.87 - 1.16]	[0.82 - 1.08]	[1.10 - 1.25]	[1.05 - 1.19]
Optimism	1.41***	1.01	1.31***	1.14***
	[1.24 - 1.61]	[0.88 - 1.15]	[1.24 - 1.39]	[1.07 - 1.21]
Positive Character Actions	0.90	1.07	1.09**	1.05
	[0.75 - 1.09]	[0.89 - 1.28]	[1.02 - 1.16]	[0.98 - 1.13]
Engagement with Job	1.12	0.90	1.24***	1.00
	[0.96 - 1.30]	[0.77 - 1.04]	[1.17 - 1.31]	[0.95 - 1.06]
Inclusion	1.51***	1.42***	1.59***	1.38***
	[1.31 - 1.74]	[1.23 - 1.64]	[1.50 - 1.68]	[1.30 - 1.47]
Organizational Trust	1.19**	1.39***	1.22***	1.25***
	[1.00 - 1.42]	[1.18 - 1.64]	[1.15 - 1.29]	[1.18 - 1.32]
Friendship	1.47***	1.13*	1.34***	1.15***
	[1.28 - 1.68]	[0.98 - 1.30]	[1.26 - 1.42]	[1.08 - 1.22]
Family Satisfaction	1.38***	1.35***	1.31***	1.27***
	[1.21 - 1.58]	[1.19 - 1.54]	[1.24 - 1.39]	[1.21 - 1.34]
Family Support	1.37***	1.31***	1.15***	1.14***
	[1.19 - 1.59]	[1.14 - 1.50]	[1.08 - 1.22]	[1.08 - 1.21]
Spirituality	1.23***	1.05	1.22***	0.95*
	[1.06 - 1.44]	[0.90 - 1.22]	[1.15 - 1.30]	[0.89 - 1.01]
witnessed deaths during deployment	1.63***	3.18***	1.34***	2.48***
	[1.51 - 1.76]	[2.98 - 3.39]	[1.29 - 1.39]	[2.40 - 2.56]
discharged weapon during deployment	0.91**	1.57***	0.91***	1.41***
	[0.83 - 1.00]	[1.46 - 1.68]	[0.86 - 0.95]	[1.36 - 1.47]
wounded or in danger during deployment	2.22***	8.16***	2.17***	6.10***
	[2.04 - 2.42]	[7.41 - 8.97]	[2.09 - 2.26]	[5.87 - 6.34]
miss combat exposure responses	1.41***	4.57***	1.28***	3.60***
	[1.24 - 1.61]	[4.00 - 5.23]	[1.21 - 1.37]	[3.40 - 3.81]
**Demographic characteristics**				
Age	1.00	1.01*	1.00*	1.01***
	[0.99 - 1.01]	[1.00 - 1.01]	[1.00 - 1.01]	[1.01 - 1.02]
Female	1.42***	1.97***	1.30***	1.65***
	[1.29 - 1.56]	[1.80 - 2.15]	[1.24 - 1.37]	[1.57 - 1.73]
Black	1.31***	1.18***	1.23***	1.15***
	[1.21 - 1.43]	[1.09 - 1.27]	[1.18 - 1.29]	[1.11 - 1.20]

Appendix Table 2 (Continued)

	Restricted Sample		Whole Sample	
Outcome=	Depression	PTSD	Depression	PTSD
Hispanic	1.00	1.05	1.02	1.07***
	[0.91 - 1.11]	[0.96 - 1.14]	[0.96 - 1.07]	[1.03 - 1.12]
Asian	1.18**	1.05	1.07	0.95
	[1.02 - 1.37]	[0.92 - 1.19]	[0.98 - 1.17]	[0.88 - 1.03]
Other minority	0.80	0.94	1.02	1.15***
	[0.54 - 1.19]	[0.69 - 1.28]	[0.90 - 1.14]	[1.05 - 1.27]
At least college degree	0.88**	0.90**	0.87***	0.89***
	[0.78 - 1.00]	[0.81 - 1.00]	[0.81 - 0.94]	[0.83 - 0.94]
Married	1.05	1.06	1.06**	1.12***
	[0.95 - 1.17]	[0.96 - 1.16]	[1.01 - 1.12]	[1.07 - 1.17]
Divorced	0.98	1.05	1.09	1.12**
	[0.74 - 1.29]	[0.84 - 1.32]	[0.97 - 1.23]	[1.02 - 1.24]
Have kids	0.99	1.09	0.99	1.05**
	[0.88 - 1.12]	[0.98 - 1.20]	[0.94 - 1.05]	[1.00 - 1.10]
**Service characteristics**				
AFQT scores (ref group <=30)				
AFQT scores 31-49	0.86	0.82	1.02	0.93
	[0.60 - 1.22]	[0.60 - 1.10]	[0.93 - 1.13]	[0.86 - 1.01]
AFQT scores 50-64	0.89	0.77*	0.98	0.85***
	[0.62 - 1.27]	[0.57 - 1.04]	[0.89 - 1.09]	[0.78 - 0.93]
AFQT scores 65-92	0.87	0.67***	0.98	0.73***
	[0.61 - 1.24]	[0.49 - 0.90]	[0.89 - 1.09]	[0.67 - 0.79]
AFQT scores 93-100	0.81	0.55***	0.89*	0.58***
	[0.56 - 1.17]	[0.40 - 0.75]	[0.79 - 1.01]	[0.52 - 0.65]
MOS categories (ref group is Combat Arms)				
Aviation	1.32***	1.40***	1.13***	1.08
	[1.14 - 1.53]	[1.21 - 1.62]	[1.03 - 1.24]	[0.98 - 1.19]
Combat service	1.19***	1.48***	1.15***	1.27***
	[1.09 - 1.30]	[1.37 - 1.60]	[1.10 - 1.21]	[1.22 - 1.32]
Service support	0.99	1.17***	1.00	1.07**
	[0.90 - 1.10]	[1.07 - 1.28]	[0.95 - 1.06]	[1.01 - 1.12]
Other occupation	0.97	1.27***	1.04*	1.18***
	[0.88 - 1.07]	[1.17 - 1.38]	[0.99 - 1.09]	[1.13 - 1.22]
Rank (ref group is E4)				
Rank E1-E2	2.07***	2.35***	1.75***	2.28***
	[1.79 - 2.40]	[2.02 - 2.74]	[1.58 - 1.93]	[2.15 - 2.42]
Rank E3	1.18***		1.18***	0.91***
	[1.10 - 1.27]		[1.12 - 1.24]	[0.87 - 0.96]
Rank E5			0.87***	0.78***
			[0.84 - 0.92]	[0.74 - 0.83]
Rank E6			0.73***	0.96**
			[0.69 - 0.78]	[0.92 - 0.99]
Rank E7			0.66***	0.96**
			[0.61 - 0.72]	[0.92 - 0.99]
Cohort (ref group is joined 2000 or earlier)				
joined Army b/w 2001-2008			1.11***	1.51***
			[1.05 - 1.17]	[1.36 - 1.67]

Appendix Table 2 (Continued)

	Restricted Sample		Whole Sample	
Outcome=	Depression	PTSD	Depression	PTSD
joined Army 2009 and onward			0.99	1.00
			[0.92 - 1.05]	[0.96 - 1.05]
deployed prior to GAT			0.94***	1.04*
			[0.90 - 0.98]	[1.00 - 1.08]
**Baseline health assessment**				
pre-deployment assessment available	0.95	1.10*	0.81***	1.04*
	[0.84 - 1.07]	[0.99 - 1.23]	[0.77 - 0.84]	[1.00 - 1.08]
sought psychological health counseling pre-deployment	2.46***	2.35***	2.34***	2.28***
	[2.12 - 2.85]	[2.02 - 2.74]	[2.20 - 2.50]	[2.15 - 2.42]
Constant	0.03***	0.01***	0.81***	0.02***
	[0.02 - 0.05]	[0.01 - 0.02]	[0.03 - 0.04]	[0.02 - 0.02]
Sample size	62,913	62,754	223,492	222,846

*** p<0.01, ** p<0.05, * p<0.1

Note:

Depression: screened positive for depression symptoms based on 2-item Patient Health Questionnaire

PTSD: screen positive for PTSD symptoms based on the Primary Care PTSD screen
